# Evaluating Transmission Paths for Three Different *Bartonella* spp. in *Ixodes ricinus* Ticks Using Artificial Feeding

**DOI:** 10.3390/microorganisms9050901

**Published:** 2021-04-22

**Authors:** Nina Król, Nina Militzer, Elisa Stöbe, Ard M. Nijhof, Martin Pfeffer, Volkhard A. J. Kempf, Anna Obiegala

**Affiliations:** 1 Institute of Animal Hygiene and Veterinary Public Health, University of Leipzig, 04103 Leipzig, Germany; nina.krol@vetmed.uni-leipzig.de (N.K.); elisastoebe@icloud.com (E.S.); pfeffer@vetmed.uni-leipzig.de (M.P.); 2 Institute of Parasitology and Tropical Veterinary Medicine, Freie Universität Berlin, 14163 Berlin, Germany; nina.militzer@fu-berlin.de (N.M.); ard.nijhof@fu-berlin.de (A.M.N.); 3 Institute for Medical Microbiology and Infection Control, University Hospital, Goethe University, 60596 Frankfurt am Main, Germany; volkhard.kempf@kgu.de

**Keywords:** *Bartonella schoenbuchensis*, *Bartonella henselae*, *Bartonella grahamii*, *Ixodes ricinus*, nymphs, females, vector, transstadial transmission, transovarial transmission, artificial feeding

## Abstract

Bartonellae are facultative intracellular alpha-proteobacteria often transmitted by arthropods. *Ixodes ricinus* is the most important vector for arthropod-borne pathogens in Europe. However, its vector competence for *Bartonella* spp. is still unclear. This study aimed to experimentally compare its vector competence for three *Bartonella* species: *B. henselae*, *B. grahamii*, and *B. schoenbuchensis*. A total of 1333 ticks (1021 nymphs and 312 adults) were separated into four groups, one for each pathogen and a negative control group. Ticks were fed artificially with bovine blood spiked with the respective *Bartonella* species. DNA was extracted from selected ticks to verify *Bartonella*-infection by PCR. DNA of *Bartonella* spp. was detected in 34% of nymphs and females after feeding. The best engorgement results were obtained by ticks fed with *B. henselae*-spiked blood (65.3%) and *B. schoenbuchensis* (61.6%). Significantly more nymphs fed on infected blood (37.3%) molted into adults compared to the control group (11.4%). *Bartonella* DNA was found in 22% of eggs laid by previously infected females and in 8.6% of adults molted from infected nymphs. The transovarial and transstadial transmission of bartonellae suggest that *I. ricinus* could be a potential vector for three bacteria.

## 1. Introduction

Bartonellae are Gram-negative α-proteobacteria that can cause mild to life-threatening symptoms in humans depending on the causing *Bartonella* species [1]. Despite their virulence and worldwide distribution, bartonellae are among the bacterial pathogens that are considered neglected regarding diagnostic investigation and awareness of practitioners [2]. According to their phylogenetic relationship and their pathogenicity factors, bartonellae can be classified into four deep-branching lineages of eubartonellae and two additional ancestral *Bartonella* species, *B. apis* and *B. tamiae* [3]. Lineage 1 is considered the most virulent *Bartonella* group and contains *B. bacilliformis*, which may cause life-threatening infections in humans [4]. The other three *Bartonella* lineages are considered less pathogenic and evolutionarily more advanced. Lineage 2 contains bartonellae that are harbored by domesticated and wild ruminants. Lineages 3 and 4 each contain a large number of different *Bartonella* species with various mammalian reservoir host species. The potential reservoir hosts of lineage 3 are primarily rats, cats, dogs, and foxes, whereas the main potential reservoirs of lineage 4 are mainly small mammals such as voles and mice [5,6].

In general, arthropods such as fleas, lice, deer keds, and sand flies are essential for the transmission of their associated *Bartonella* species [6,7,8]. However, the vector function of the most common tick species in Europe, *Ixodes ricinus* (the castor bean tick), is considered controversial for *Bartonella* spp. There is some evidence that *I. ricinus* can harbor various *Bartonella* species, and one study investigated the experimental acquisition and transmission of the zoonotic *B. henselae* (lineage 4) by *I. ricinus* [9]. Further, epidemiological studies showed that *I. ricinus* harbored ruminant-associated bartonellae (lineage 2) such as *B. schoenbuchensis* that can cause fatigue and fever in humans [10,11,12]. Additionally, DNA of small mammal-associated bartonellae (lineage 4), such as *B. grahamii,* has been detected in *I. ricinus* ticks [13]. It has however not been experimentally determined if *I. ricinus* may be a vector for *Bartonella* spp. other than *B. henselae*. Moreover, it is not known if the combination of the respective pathogenicity factors of different *Bartonella* species may have an influence on the development of *I. ricinus* and its vector competence. Liu et al. [14] showed a higher weight of *I. ricinus* nymphs after being fed on *B. henselae*-infected blood. This could be linked to an upregulation of a tick serine protease inhibitor, which was caused by *B. henselae* infection.

In order to adhere to the 3R principle (reduce, replace, refine) for humane animal research, laboratory tick feeding methods based on artificial membrane feeding have been established and used to study tick biology and conduct experimental infections with tick-borne pathogens [15,16,17,18,19,20,21,22,23].

As the vector function of *I. ricinus* is still unknown for many *Bartonella* species, the aims of this study were: (i) to infect different stages of *I. ricinus* ticks by artificial feeding with three zoonotic *Bartonella* species (*B. grahamii*, *B. henselae*, and *B. schoenbuchensis*) (ii) to evaluate tick developmental proportions and engorgement weight following artificial infection with the respective *Bartonella* species; and (iii) to evaluate the potential vector competence of *I. ricinus* with regard to transovarial and transstadial transmission for these three *Bartonella* species.

## 2. Materials and Methods

### 2.1. Cultivation of Bartonella colonies and Optical Density Measurement

Laboratory colonies of *B. henselae* Marseille, *B. schoenbuchensis* DSMZ 13525, and *B. grahamii* ATCC700132 provided by the conciliar laboratory for *Bartonella* in Germany (V.A.J. Kempf) were kept under sterile conditions on 7% sheep blood Columbia Agar (Henry Schein Medical GmbH, Berlin, Germany) at 37 °C, 70% relative humidity (RH), and 5% CO_2_ atmosphere. For spiking blood (see below) for tick feeding (every 24 h) or inoculating fresh blood agar plates (every 3–4 days), colonies from one blood plate were suspended in 1 mL PBS (Dulbecco’s phosphate buffered saline, modified without calcium chloride and magnesium chloride; Sigma-Aldrich Chemie GmbH, Taufkirchen, Germany). To estimate the concentration of bacteria in PBS suspension, the optical density (OD) thus the number of colony-forming units (CFU) per mL was measured spectrophotometrically using the NanoPhotometer NP80 (IMPLEN, Munich, Germany) with 1 OD resulting in approximately 5 × 10^8^ CFU.

### 2.2. Preparation of Blood for Experimental Tick Infection

Fresh heparinized (50 I.E./mL) bovine blood was purchased (ACILA Dr. Weidner GmbH, Weiterstadt, Germany) and supplemented with 2 g of glucose (WDT, Garbsen, Germany) per liter of blood. The blood was used for up to seven days and stored at 6 °C. Gentamicin (5 µg/mL) was added to the blood 24 h before tick feeding. The respective *Bartonella* species (1 µL of 10^9^ CFU/mL of PBS per 1 mL blood) and ATP (51 mg/mL) were added shortly before each use.

### 2.3. Ticks

*Ixodes ricinus* nymphs, females, and males chosen for this experiment were mostly commercially obtained (Insect Services, Berlin, Germany). These ticks were 20–56 weeks after molting when entering the experiment. Nymphs were also provided by the Institute of Parasitology and Tropical Veterinary Medicine, FU Berlin. These ticks were 21–26 weeks after molting. All ticks were laboratory-bred and direct ancestral ticks were tested previously negative for common tick-borne pathogens such as *Babesia*, *Borrelia,* and *Rickettsia* spp. Until feeding, *I. ricinus* ticks were kept at 20 °C and 80% RH in a 15:9 h light–dark regime in an incubator (Typ MK (KL) 600, FLOHR Instruments, Nieuwegein, the Netherlands). One week prior to the experiment with adult ticks, females and males were put together in glass tubes (10–15 females and 10–15 males per tube). In each feeding experiment, ticks were divided into four groups, one for each pathogen and a negative control group. Ticks chosen for the negative control group and the *Bartonella*-infected groups derived from the same parental ticks. Further, they were fed under the same conditions as the control group before the experiment. As the control group was always negative, we consider that all ticks were negative before the experiment.

### 2.4. Preparation of Tick Feeding Units, Tick Feeding and Infection with Bartonella spp. via Artificial Feeding

**Figure 1:**
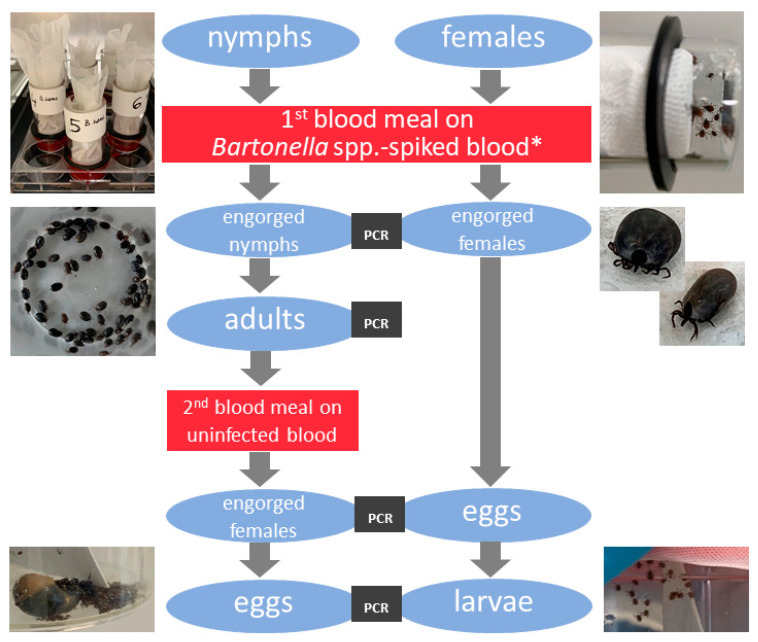
Study design of infecting *Ixodes ricinus* ticks with *Bartonella* spp. using the artificial feeding (* blood for negative control groups was spiked with PBS).

The study design is shown in Figure 1. Tick feeding units (FUs) made of glass tubes (size of “big” FU: 32 mm × 2.8 mm × 65 mm for adults and “small” 20 mm × 2.5 mm × 40 mm for nymphs; Neubert Glas, Geschwenda, Germany) sealed with silicone membranes were prepared as described previously [20,22]. The membranes were reinforced either by goldbeater’s skin (Altenburger Pergament & Trommelfell GmbH, Altenburg, Germany) for nymphs or by lens cleaning paper (Whatman, Maidstone, UK) for adult ticks. Membranes were left to polymerize for at least 24 h at room temperature. The thickness of the membranes was measured using the Inductive Dial Comparator 2000 (Mahr, Göttingen, Germany). Membranes with a thickness of 50–70 µm or 80–120 µm were used for nymphs and adults, respectively. The silicone membranes were glued to the glass tubes using silicone glue (Elastosil E41, Wacker, Munich, Germany). FUs were left for 24 h to harden and checked for leakage by immersion in 70% ethanol for at least 15 min. A net of 2 cm × 2 cm fiberglass (Drahtwaren-Driller GmbH, Freiburg, Germany) was glued (using Elastosil E41) on the inside of the big FUs, which were additionally autoclaved before usage. For all FUs, a sheep hair extract [20] was applied (0.348 mg/small FU and 0.525 mg/big FU) to the inner side of the membrane 2–5 h (including 30 min on a hot plate at 45 °C) before ticks were put in the feeding units. The small FU contained 20–25 nymphs and the big FU contained 10–15 females together with the same number of males. The units were sealed with punctured plastic lids and fine mesh (big FUs) or sponges (small FUs). Supplemented blood was prewarmed at 38 °C in a water bath before adding it into 6-well (3.1 mL per well for adults) or 12-well plates (1 mL per well for nymphs). FUs with ticks were fixed with rubber rings (Hansa Armaturen GmbH, Stuttgart, Germany) and immersed in blood in the wells. The well plates were placed on a hot plate XH-2002 (C & A Scientific Co., Sterling, VA, USA) during blood changes. *Bartonella* spp. suspensions or PBS (for negative control groups) were added to each blood meal starting 24 h after the beginning of the tick feeding. Blood was replaced with fresh blood in new wells twice a day (every 10–14 h). Before replacing FUs into fresh blood, the outer surfaces of FUs and membranes were rinsed with preheated sterile 0.9% NaCl (38 °C). FUs were opened every 24 h, starting on the 3rd day p.i., in order to remove feces, detached and dead ticks. Well plates with FUs were then placed on hot plates (T 37 °C; Hot Plate 062, Labotect, Göttingen, Germany) and a shaker (100 rpm, IKA MTS 2/4 digital, Staufen, Germany) in the same climatic conditions as described for ticks in the chapter above. The feeding experiment lasted 15 days for adults and 8 days for nymphs or until natural detachment of the ticks.

In the case of visible fungal contamination, FUs were placed in 10,000 units/mL of nystatin ready-made solution (Sigma Aldrich) for 5 min and cleaned with 0.9% NaCl before reimmersing into fresh blood. Additionally, blood samples from used wells were taken twice a day to confirm the presence of *Bartonella* spp.

Detached engorged ticks were weighed (females individually, nymphs in groups) and stored in plastic containers with punctured lids and netting (adults) or in glass tubes with sponge plugs (nymphs) in a desiccator with saturated K_2_SO_4_ solution, providing a relative humidity of 95–97%, at room temperature and darkness until molting, oviposition or death.

Adults that molted from nymphs that previously fed on infected blood (first feeding) were fed on uninfected blood until natural detachment (Figure 1). Engorged females were weighed individually and nymphs in pools of 50 with a precision scale (R-160-P, Sartorius, Göttingen, Germany). Potentially infected females were paired with uninfected males.

The feeding experiments were performed in the spring and autumn of 2019 and 2020. feeding system.

### 2.5. Bartonella Detection in Ticks and Blood via PCR

In order to confirm *Bartonella* spp. transmission, 10% of freshly engorged ticks (showing the lowest chances for further development; for nymphs: no reaction to CO_2_ and for females additionally low engorgement weight), adults molted from potentially infected nymphs, eggs and larvae from potentially infected females, and eggs of females molted from potentially infected nymphs, and blood samples after feeding were examined for the presence of *Bartonella* DNA by PCR. DNA was extracted individually from nymphs and females; eggs and larvae deriving from engorged females were examined in pools per single female that laid them. DNA extraction was performed with the QIAamp DNA Mini Kit (Qiagen, Hilden, Germany) as recommended in the manufacturer’s protocol for blood respectively for tissue. To detect DNA of *Bartonella* spp. in ticks after feeding on infected blood, a real-time PCR assay targeting a 301-bp region of the *ssrA* gene was used [24]. Further, to confirm transstadial and transovarial transmission of bartonellae, the 16S–23S rRNA intergenic spacer region was targeted as described previously [25] with extended cycling conditions to 45. The PCR for molted ticks, eggs, and hatched larvae was run twice, with the second reaction performed on purified products from the first round (Macherey-Nagel GmbH & Co. KG, Düren, Germany). Subsequently, electrophoresis was performed and analyzed using a UVP GelSolo (Analytik Jena GmbH, Jena, Germany).

### 2.6. Recultivation of Bartonella from Tick Gut and Salivary Glands

After being fed on *Bartonella*-spiked blood, nymphs molted into potentially infected adults. A selected number of 16 female adults (4 per pathogen and 4 for the negative control) were fed with uninfected blood. After 60 h, blood after feeding and females were removed from the units and their salivary glands and their midgut were dissected. Blood after feeding and the removed salivary glands and midgut were incubated separately in 1 mL Schneider Drosophila medium (Th. Geyer GmbH, Renningen, Germany) at 35 °C in an atmosphere of 5% CO_2_ for 48 h. Finally, 10 μL of each incubated sample were placed on Columbia Agar plates and incubated as described above.

### 2.7. Statistical Analyses

Statistical analyses were conducted using IBM SPSS statistics (version 25). Confidence intervals (95% CI) for the prevalence rates and chi-squared and Fisher’s exact tests were used to compare the engorgement rates and pathogen transmission in ticks regarding the respective *Bartonella* species. The engorgement weights of females were compared using the one-tailed Mann–Whitney U test. The significance threshold was set at *p* = 0.05.

## 3. Results

### 3.1. Tick Feeding and Ticks Development

In general, 1333 *I. ricinus* ticks were used in this experiment, 1021 nymphs and 312 adults (156 females and 156 males) (Table 1).

In total, 587 out of 1021 nymphs engorged and detached leading to the engorgement rate of 57.5% (95% CI: 54.4–60.5%), and 111 out of 156 females (71.2%; 95% CI: 65.6–77.7%), (Table 1). In the case of nymphs, ticks from the negative control group obtained the lowest engorgement rate (46%; 95% CI: 37.97–54.32) and the highest rate (65.3%; 95% CI: 59.65–70.53) was observed for nymphs feeding on *B. henselae*-spiked blood (χ^2^ = 15.72, df = 3, *p* = 0.001). In contrast to nymphs, females from the negative control reached the highest engorgement rate (90%; 95% CI: 68.68–98.43) and the lowest rate (57.7%; 95% CI: 44.18–70.14) was noted for females fed on *B. grahamii*-infected blood (χ^2^ = 13.355, df = 3, *p* = 0.004).

The mean weight of nymphs was 2.94 mg and for females 170.74 mg (SD = 82.634) (Table 1). The average mean weight of nymphs potentially infected with *B. schoenbuchensis* (3.21 mg) was the highest and the lowest for nymphs fed on *B. grahamii*-infected blood (2.71 mg). In the case of females, the highest mean weight was noted for ticks from the negative control (189.57 mg, SD = 94.892) and the lowest for females exposed to infection with *B. grahamii* (154.66 mg, SD = 81.99). However, there were no significant differences in the mean weight between females from the control group and those potentially infected (U = 732.5, Z = −0.654, *p* = 0.513).

The developmental success (Table 2) for nymphs fed on blood infected with three different *Bartonella* spp. showed no statistical differences: 195 of 523 engorged nymphs (37.3%; 95% CI: 33.25–41.51) successfully molted into adults (97 males and 98 females) (χ^2^ = 0.602, df = 2, *p* = 0.74). However, nymphs from the control group reached significantly lower molting rates than nymphs feeding on infected blood and only 5 out of 44 (11.4%; 95% CI: 4.5–24.43) uninfected engorged nymphs molted into adults (2 males, 3 females), (χ^2^ = 12.56, df = 3, *p* = 0.006).

The highest rate for engorged females laying eggs (Table 2) was noted for females feeding on *B. schoenbuchensis*-spiked blood (91.7%; 95% CI: 77.43–97.87) and significantly the lowest for ticks feeding on *B. grahamii*-infected blood (50%; 95% CI: 33.15–66.85), (χ^2^ = 14.443, df = 3, *p* = 0.002). For larvae, there were no significant differences between those that hatched from females fed on infected or non-infected blood. In general, larvae hatched from eggs laid by 45% (95% CI: 34.58–55.88) of females (χ^2^ = 2.051, df = 3, *p* = 0.562).

### 3.2. Bartonella spp. Detection in Ticks

#### 3.2.1. *Bartonella* spp. Acquisition by Ticks from a Blood Meal 

*Bartonella* DNA was detected in all infected blood samples taken after tick feeding. *Bartonella* DNA was detected in 34.2% (95% CI: 26.28–43.04) (χ^2^ = 4.023, df = 2, *p* = 0.134) of the fed ticks and was observed in both nymphs and adults for all three pathogens (Table 3). There were no statistical differences regarding the respective *Bartonella* species in the acquisition from a blood meal directly to nymphs (χ^2^ = 3.844, df = 2, *p* = 0.146) or females (χ^2^ = 2.493, df = 2, *p* = 0.286). Further, there were no significant differences in bartonellae transmission between nymphs and females (*p* = 0.666 for *B. grahamii*, *p* = 0.3 for *B. henselae*, and *p* = 0.775 for *B. schoenbuchensis*). No amplified fragment was detected in ticks from negative control groups (*n* = 20) fed on blood supplemented with PBS only, and in the fed blood itself.

#### 3.2.2. *Bartonella* spp. Transstadial and Transovarial Transmission

DNA of *Bartonella* was detected in 10 adults molted from 116 potentially infected nymphs (8.6%; 95% CI: 4.59–15.3). Interestingly, the transstadial transmission of *B. schoenbuchensis* was significantly the highest (18.2%; 95% CI: 8.23–34.77) compared to both of the other pathogens (χ^2^ = 6.123, df = 2, *p* = 0.046) (Table 4).

The transmission of bartonellae from females fed on infected blood (*n* = 50) to the next generation (eggs and larvae) was observed also for all three pathogens, however without statistical differences (χ^2^ = 0.416, df = 2, *p* = 0.812) and was detected in 22% of the eggs and larvae (95% CI: 12.59–35.41) of potentially infected females.

The transovarial transmission of pathogens was also observed in the eggs of three females that were potentially infected as nymphs for all three *Bartonella* species (one female per pathogen).

Recultivation of *Bartonella* spp. from the tick gut and tick salivary glands from all females and the blood after feeding failed due to fast overgrowth of accompanying flora.

## 4. Discussion

The vector competence of *I. ricinus* ticks for three different *Bartonella* species, *B. grahamii, B. henselae,* and *B. schoenbuchensis* was experimentally analyzed under laboratory conditions in the current study.

Previously, artificial feeding was used for experiments with different tick species, mostly *I. ricinus*, *I. scapularis*, *Dermacentor reticulatus*, and *Rhipicephalus sanguineus* [15,16,18,22,23,26,27,28,29]. In total, 57.5% of nymphs and 71.2% of females from the current study engorged, which is in line with previous data from artificial feeding on non-infected blood (47.7% and 80.7%, respectively) [19,30]. The mean weight of nymphs in our study (2.71–3.21 mg) corresponds to the mean weight of nymphs (2.8 mg) fed in the same artificial feeding system [31]. In our experiment, there were no significant differences in the mean weights of females fed on *Bartonella*-spiked blood (166.58 mg) and those from the negative control group (189.57 mg). The mean weight of females (170.74 mg) from this study was comparable with the weight of females fed on *Bab. divergens* (161 mg) [29], however, lower compared to a study on non-infected blood (217 mg) [19]. The proportion of females that successfully oviposited (72.1%) was also in line with previous studies (33.3–72%) [9,19,28,29]. The molting success for infected nymphs was consistent with another study for *Bab. venatorum* (formerly EU1) (32.3%) [28] but lower compared to a control group feeding on the same artificial feeding system (54%) [32].

*Bartonella tamiae,* which was described as the causative agent for febrile symptoms in patients from Thailand, is the only zoonotic *Bartonella* species that is thought to be mainly transmitted by ticks (e.g., *I. vespertilionis* and *Haemaphysalis* spp.) [3,33]. Even though there are frequent reports about the detection of *Bartonella* DNA in ticks collected from the field, the vector role of ticks has not been elucidated for many different *Bartonella* species [34,35,36]. Furthermore, laboratory experiments with *Bartonella* spp. in ticks are scarce and mainly focused on *B. henselae* [26,30,37,38]. *Bartonella henselae* is ubiquitous and the zoonotic causative agent for the cat-scratch disease primarily evoking regional lymphadenopathy in humans [39]. While being mainly harbored by cats and transmitted by cat fleas (*Ctenocephalides felis*), this pathogen was also detected in questing ticks collected in nature [40]. Moreover, *B. henselae* was detected in ticks collected from cows, dogs, and humans, which was associated with clinical symptoms such as asthenia and headache [36,41,42,43]. As the transstadial transmission of *B. henselae* in *I. ricinus* was previously experimentally shown, our study included this pathogen as a confirmatory reference group [9]. In the current study, we confirmed that *B. henselae*-DNA can be transmitted transstadially from nymphs to adults [9]. Further *B. henselae*-positive females laid eggs in which *Bartonella* DNA was also detected, suggesting that transovarial transmission in *I. ricinus* may occur. Contrarily, a study by Cotté et al. suggested a lack of transovarial transmission for *B. henselae* in *I. ricinus* ticks, but the eggs from only nine females were tested [9]. Contamination cannot be entirely ruled out in our study, as eggs were not decontaminated before DNA extraction. Nonetheless, the transovarial transmission seems likely as hatched larvae also tested positive. In the current study, the engorgement rates for females (64.3%) and nymphs (65.3%) fed on *B. henselae*-infected blood were very similar and also similar compared to another study examining nymphs (62.3%) after *B. henselae*-infection [9]. A recent study showed that the engorgement weight of nymphs may be a predictive value concerning the future sex the nymphs develop into [44]. Lighter nymphs supposedly develop into male adults while heavier nymphs develop more likely into females. The mean weight for nymphs molting into adults was higher for *B. henselae* compared to the negative control and *B. grahamii*-infected blood. Nonetheless the ratio of developed males and females was almost alike compared to *B. grahamii* and the control group. The engorgement rate and the mean weight of females after feeding were significantly lower compared to females fed on non-infected blood in the current study. Liu et al. showed similar tendencies for *I. ricinus* comparing engorgement rates and mean weight after feeding on non-infected and *B. henselae*-infected blood [30]. However, another study by Liu et al. showed an upregulation of an *I. ricinus* serine proteinase inhibitor protein associated with *B. henselae* infection using a transcriptomic approach. Silencing the expression of this protein by RNA interference led to a lighter mean engorgement weight and a lower bacterial load in ticks [14].

Domesticated and wild ruminants are known to be reservoirs for *B. schoenbuchensis,* which is usually transmitted by deer keds (*Lipoptena cervi*) [45]. Infections with *B. schoenbuchensis* may lead to deer ked dermatitis in humans. There is even a report of clinical symptoms in a human with *B. schoenbuchensis* infection that might be associated to previous tick exposure [11]. To the authors’ knowledge, experimental infection of ticks with *B. schoenbuchensis* has never been conducted before. Ticks feeding on *B. schoenbuchensis*-infected blood performed similarly to ticks fed on *B. henselae*-infected blood concerning the engorgement rate and their detachment weight. The ratio of nymphs developing into females was however higher compared to both of the other *Bartonella* spp. and the control group. Furthermore, it was remarkable that the rate of oviposition was higher for ticks fed on *B. schoenbuchensis*-blood (91.7%) compared to *B. henselae*- (74%) and *B. grahamii*-infected blood (50%). Further, the prevalence of transstadial transmission (18.2%) was significantly higher in ticks previously fed on *B. schoenbuchensis* compared to *B. grahamii* and *B. henselae* (7.7% and 1.3%, respectively). Epidemiological studies showed also higher prevalence rates for *B. schoenbuchensis* in *I. ricinus* ticks compared to other *Bartonella* species [12,34].

*Bartonella grahamii* is mostly associated with small mammals such as bank voles (*Clethrionomys glareolus*) and field voles (*Microtus agrestis*) being the main reservoirs [46]. Fleas associated with these small mammals serve as vectors. However, *B. grahamii* can also be transmitted to humans by cat scratches [47] causing similar symptoms as *B. henselae*. *Ixodes ricinus* ticks collected from nature or from small mammals tested positive for this pathogen in earlier investigations [13,48]. In the current study, ticks fed on *B. grahamii*-infected blood performed the worst concerning the engorgement rate and the detachment weight after feeding compared to the other two pathogens. Further, we observed that some ticks fed on *B. grahamii*-infected blood died during tick feeding. However, the prevalence for *B. grahamii* in eggs laid by infected females was comparable to rates for *B. henselae* and *B. schoenbuchensis,* and in molted adults it was higher than *B. henselae* and lower than *B. schoenbuchensis*. A former study showed that the feeding time or engorgement status of *I. ricinus* is not influenced by the species of the blood donor [30]. Unlike *B. henselae* and *B. schoenbuchensis*, *B. grahamii* has never been naturally detected in bovine blood [36,49]. The lacking adaption of *B. grahamii* to bovine blood may be a reason for the differing results concerning tick development in comparison to both other *Bartonella* spp. Moreover, all selected *Bartonella* spp. in this study have a different combination of pathogenicity factors, which may also be a reason for differing results in ticks [3]. However, the influence of pathogenicity factors in vectors has not been examined yet. While *B. henselae* and *B. grahamii* belong phylogenetically to the same clade (lineage 4), *B. schoenbuchensis* is more distinct (lineage 2) in regard to phylogeny, main vectors, and reservoir hosts [3]. A previous study on host–pathogen coevolution showed a significant coevolutionary fit and patterns of cospeciation with minimal host switching for *Bartonella* spp. [50]. Yet, a similar study for vector–pathogen associations is missing but evolutionary adaptation may be a reason for the differing outcome concerning the three examined *Bartonella* species.

The recultivation of *Bartonella* spp. from infected *I. ricinus* failed due to a fast proliferation of accompanying flora on the agar plates. This is why the described transovarial and transstadial transmission in ticks can only be reliably described for *Bartonella*-DNA and not necessarily for the viable bacteria. Nonetheless, the current study points out that *I. ricinus* should be regarded as a potential vector for the examined *Bartonella* species.

Future studies by our group will focus on the bacterial transmission to mammal models to identify the onset of symptoms/disease and to verify the viability of the detected *Bartonella* spp. in blood and ticks.

## 5. Conclusions

Our study showed that transstadial and transovarial transmission for *B. grahamii*, *B. henselae*, and *B. schoenbuchensis* may occur in *I. ricinus* ticks. Even though transstadial and transovarial prevalence rates were low to moderate (1.3–26.1%) these results suggest that *I. ricinus* is a possible vector for these *Bartonella* species. The success of tick engorgement, development, and *Bartonella* prevalence in *I. ricinus* might depend on the respective *Bartonella* sp. Further research is needed to unveil the mechanisms leading to the described differing results.

## Figures and Tables

**Table 1:** Number of *Ixodes ricinus* ticks used in the artificial feeding experiment fed on blood spiked with different *Bartonella* species and PBS (negative control groups), their engorgement rates, and mean weight.

Life Stage	Blood Meal Spiked with	No. of Feeding Ticks	Engorgement Proportion (%, (no. of Engorged))	Mean Weight after Feeding (mg, (SD) *)
Nymphs	*B. grahamii*	300	54.6 (*n* = 164)	2.71
	*B. henselae*	291	65.3 (*n* = 190)	3.01
	*B. schoenbuchensis*	291	58.1 (*n* = 169)	3.21
	PBS	139	46.0 (*n* = 64) ^a^	2.82
	total	1021	57.5 (*n* = 587)	2.94
Females	*B. grahamii*	52 (+52 males)	57.7 (*n* = 30) ^b^	154.7 (±81.99)
	*B. henselae*	42 (+42 males)	64.3 (*n* = 27)	175.6 (±85.83)
	*B. schoenbuchensis*	42 (+42 males)	85.7 (*n* = 36)	168.1 (±75.80)
	PBS	20 (+20 males)	90 (*n* = 18)	189.6 (±94.89))
	total	156 (+156 males)	71.2 (*n* = 111)	170.7 (±82.63)

No.—number; SD (±)—standard deviation; * SD values were calculated only for females as nymphs were weighed in pools; ^a^ the lowest engorgement rate for nymphs (χ^2^ = 15.72, df = 3, *p* = 0.001); ^b^ the lowest engorgement rate for females (χ^2^ = 13.355, df = 3, *p* = 0.004).

**Table 2:** Developmental success of ticks feeding on blood spiked with *Bartonella* spp. and PBS (negative control groups).

Developmental Stages	Developmental Success for Ticks Feeding on Blood Spiked with			
	*B. grahamii*	*B. henselae*	*B. schoenbuchensis*	PBS
Engorged nymphs molting into adults (%; no. molted adults/no. engorged nymphs)	38.4 (63 (29m:34f)/164)	38.4 (73 (44m:29f)/190)	34.9 (59 (24m:35f)/169)	11.4 ^a^ (5 (2m:3f)/44)
Engorged females laying eggs (%; no. females laying eggs/no. engorged females)	50 (15/30) ^b^	74 (20/27)	91.7 (33/36)	66.7 (12/18)
Eggs molting into larvae (%; no. females producing larvae/no. females laying eggs)	33.3 (5/15)	40 (8/20)	48.5 (16/33)	58.3 (7/12)

No.—number of, m:f—ratio of males and females, which developed from engorged nymphs; ^a^ the lowest molting success for nymphs (χ^2^ = 12.56, df = 3, *p* = 0.006); ^b^ the lowest oviposition (χ^2^ = 14.443, df = 3, *p* = 0.002).

**Table 3:** PCR detection of *Bartonella* spp. transmission from the blood meal to feeding ticks.

Tick Life Stage	*Bartonella* spp. Detection (%; (no. Positive/no. Tested)) in Ticks Fed on Blood Spiked with			Total
	*B. grahamii*	*B. henselae*	*B. schoenbuchensis*	
Nymphs	17.6 (3/17)	40 (8/20)	47.6 (10/21)	36.2 (21/58)
Females	30.8 (4/13)	20 (4/20)	41.1 (12/29)	32.3 (20/62)
Total	23.3 (7/30)	30 (12/40)	44 (22/50)	34.2 (41/120)

No.—number of.

**Table 4:** Transmission success of *Bartonella* species between tick life stages.

Developmental Stage	*Bartonella* spp. Detection (%; (no. Positive/no. Tested)) in Ticks Infected with		
	*B. grahamii*	*B. henselae*	*B. schoenbuchensis*
Adults molted from potentially infected nymphs	7.7 (3/39)	1.3 (1/44)	18.2 (6/33) ^a^
Potentially infected females that produced infected eggs and larvae	18.2 (2/11)	18.6 (3/16)	26.1 (6/23)

No.—number of; ^a^ the highest transstadial transmission (χ^2^ = 6.123, df = 2, *p* = 0.046).
